# STAT3-blocked whole-cell hepatoma vaccine induces cellular and humoral immune response against HCC

**DOI:** 10.1186/s13046-017-0623-0

**Published:** 2017-11-07

**Authors:** Qiuju Han, Yaqun Wang, Min Pang, Jian Zhang

**Affiliations:** Institute of Immunopharmaceutical Sciences, School of Pharmaceutical Sciences, Shandong University, Jinan, Shandong 250012 China

**Keywords:** Tumor vaccine, STAT3, Hepatoma, Immunotherapy, Whole-cell vaccine

## Abstract

**Background:**

Whole-cell tumor vaccines have shown much promise; however, only limited success has been achieved for the goal of eliciting robust tumor-specific T-cell responses.

**Methods:**

Hepatocellular carcinoma (HCC) cells, H22 and Hepa1–6, were modified by blocking the STAT3 signaling pathway with a STAT3 decoy oligodeoxynucleotide, and the immunogenicity and possibility of using these cell lysates as a vaccine were evaluated.

**Results:**

STAT3-blocked whole HCC cell lysates inhibited tumor growth and tumorigenesis, and prolonged the survival of tumor-bearing mice. In addition, STAT3-blocked whole HCC cell lysates stimulated the activation of T cells and natural killer (NK) cells, and enhanced the infiltration of cytotoxic CD8^+^ T cells in the tumor tissues. In addition, the maturation of dendritic cells (DCs) was enhanced, which promoted the generation of immunological memory against HCC. Furthermore, secondary immune responses could be primed as soon as these immunized mice were challenged with HCC cells, accompanied by T cell and NK cell activation and infiltration. Additionally, immunization with this vaccine decreased the generation of Tregs and the production of TGF-β and IL-10. Importantly, STAT3-blocked whole HCC cell lysates prevented HCC-mediated exhaustion of T cells and NK cells, showing low expression of checkpoint molecules such as PD-1 and TIGIT on T cells and NK cells in the immunized mice.

**Conclusions:**

The newly generated STAT3-blocked whole-cell HCC vaccine has potential for cancer cell vaccination.

**Electronic supplementary material:**

The online version of this article (10.1186/s13046-017-0623-0) contains supplementary material, which is available to authorized users.

## Background

Hepatocellular carcinoma (HCC) is the most common primary liver malignancy, with high morbidity and mortality, and is the third leading cause of cancer-related death worldwide. Traditional methods to treat HCC include surgery, radiotherapy, and chemotherapy [1]. However, the efficacy of these treatments is often unsatisfactory, because of obvious side effects, ease of relapse and metastasis, and poor prognosis. Thus, the development of novel approaches for HCC treatment is urgently required. In recent years, along with the rapid development of biomolecular technology and immunology, tumor biological therapy has become a novel and effective therapeutic tool in comprehensive cancer treatment, and has become the fourth mode after surgery, chemotherapy, and radiotherapy [2].

A cancer vaccine provides proactive immunotherapy by inducing anti-tumor immune responses. To date, several HCC vaccine clinical trials have been designed based on HCC-specific tumor-associated antigens (TAAs), including alpha fetoprotein (AFP), glypican 3 (GPC3), telomerase reverse transcriptase (TERT), melanoma-associated antigen (MAGE-A), synovial sarcoma, X Breakpoint 2 (SSX-2), and New York esophageal squamous cell carcinoma 1 (NY-ESO-1) [3–5]. However, immunizations with only one or several TAAs generally fail to control overall tumor development, instead they create favorable conditions for the growth of tumor cell clones that lack the antigens present in the vaccine [3]. Recently, whole tumor cells attenuated by different kinds of treatment or mixed with various adjuvants have become the mainstream tools for application of HCC vaccines [6]. Unlike tumor-derived specific peptides, a whole tumor lysate is applicable to all patients, regardless of HLA type. Whole-cell vaccination provides multiple known and unknown TAAs to activate CD4^+^ T helper and CD8^+^ cytotoxic lymphocytes (CTL) simultaneously via the vast amount of uncharacterized and characterized T cell epitopes, decreasing the chance of tumor immune escape. A study involving approximately 1800 patients demonstrated that patients treated by whole tumor vaccination had a significantly higher objective response than patients immunized with defined tumor antigens [7].

An irradiated autologous whole tumor lysate was used to treat patients with cancer [8, 9]. However, phase III trials of whole-cell vaccines often failed to demonstrate clinical benefit [10]. One reason is the low efficiency of antigen uptake and presentation, as well as the poor immunogenicity of the tumor lysate, which cannot induce a strong anti-tumor immune response. Other explanations include immune tolerance and immunosuppression within the tumor stromal microenvironment. To overcome these defects, whole-cell tumor vaccines have been modified by overexpressing stimulatory molecules, such as fibroblast activation protein (FAP), granulocyte-macrophage colony-stimulating factor (GM-CSF), and CD86, or combined with CpG oligodeoxynucleotides (CpG ODNs), all of which conferred significant antitumor effects [11–13]. Moreover, depletion of regulatory T cells (Tregs) increases the effectiveness of tumor-cell vaccines [7].

Signal transducer and activator of transcription 3 (STAT3) is constitutively activated and overexpressed in many primary tumors, and is closely associated with tumor proliferation, angiogenesis, and immune escape [14]. Our previous findings confirmed that blocking the STAT3 signaling pathway in HCC cells inhibited proliferation and promoted the apoptosis of tumor cells. Meanwhile, the sensitivity of STAT3-blocked HCC cells to natural killer (NK) cell cytolysis was significantly enhanced. Most importantly, mice inoculated with STAT3-blocked HCC cells could effectively break tumor-induced immune tolerance, resulting in an effective anti-tumor effect [15, 16]. These results suggested that the expression of tumor antigens in HCC cells might be modified by blocking STAT3 signaling, which would enhance the immunogenicity of the HCC cells. Based on these findings, we hypothesized that STAT3-blocked HCC cells could be used as a vaccine.

To confirm this hypothesis, in the present study, we prepared a whole cell lysate of STAT3-blocked HCC cells and then evaluated it as an anti-HCC vaccine. We found that immunization with this cell lysate promoted the generation of immune memory, and induced an effective anti-HCC immune response in vivo. The results of this study suggested that blocking the STAT3 signaling pathway of HCC cells might be an efficient strategy to develop an HCC vaccine.

## Methods

### Mice and cell lines

Male BALB/c, C57BL/6 mice, and T cell-deficient nude (BALB/cA-nu) mice were obtained from Beijing HFK Bioscience (Beijing, China). All procedures were performed in accordance with the Institutional Animal Care and Use Committee Protocols of Shandong University. H22 cells (BALB/c-derived hepatoma, obtained from the Shandong Academy of Medical Sciences) were cultured in Roswell Park Memorial institute (RPMI) 1640 medium (Life Technologies BRL, Gaithersburg, MD, USA), and Hepa1–6 cells (C57BL/6-derived hepatoma, purchased from Cell Bank of Type Culture Collection of the Chinese Academy of Sciences) were cultured in Dulbecco’s modified Eagle’s medium (DMEM; Life Technologies BRL, Gaithersburg, MD), all media were supplemented with 10% fetal bovine serum (FBS; Sijiqing, Hangzhou, China).

### Reagents

The STAT3 decoy oligonucleotide (ODN) sequences were 5′-CATTTCCCGTAAATC-3′, and 5′-GATTTACGGGAAATG-3′, whereas the scrambled sequences were 5′-CATCTTGCCAATATC-3′ and 5′-GATATTGGCAAGATG-3′ [16]. These ODNs were modified with phosphorothioate, and the sense and antisense strands were annealed and purified using high performance liquid chromatography (Takara, Dalian, China). All ODNs were prepared at a concentration of 100 mM.

### Preparation of tumor cell vaccine

H22 cells or Hepa1–6 cells were transfected with STAT3 decoy or scrambled ODN using Lipofectamine 2000 (Invitrogen, Carlsbad, CA, USA), as previously described [15, 16], and then cultured in complete medium containing 10% FBS. Twenty-four hours later, the cells were inactivated through repeated cycles of liquid nitrogen freeze-thawing. After centrifugation at 1200 rpm, the cell lysates were used as HCC vaccines.

### Immunization and tumor models

Male BALB/c mice were immunized with 100 μL of lysate from 2 × 10^6^ H22 cells treated with Lipofectamine 2000, scrambled-ODN or decoy-ODN, via subcutaneous (s.c.) injection in the right flank once a week for 3 weeks. Mice injected (s.c.) with an equal volume of phosphate-buffered saline (PBS) in their right flank were defined as the control (Ctrl). One week after the last immunization, all mice were injected (s.c.) with 2 × 10^6^ H22 cells in their left flank. C57BL/6 mice were immunized as the same manner, and challenged with Hepa1–6 cells.

### Flow cytometry

Splenocytes and liver mononuclear cells were isolated as described previously [16]. Flow cytometric analysis was performed using BD FACSCalibur and FACSAria III instruments. Antibodies used in this study included fluorescein isothiocyanate (FITC)-labeled anti-mouse CD49b (DX5), anti-NK1.1, anti-CD4, and anti-CD11c; Phycoerythrin (PE)-labeled anti-mouse-CD69, anti-CD107a, anti-CD44, anti-CD86, and anti-IFN-γ; PE-cyanine 5.5-labeled anti-mouse CD3e and anti-CD8; and allophycocyanin (APC)-labeled anti-mouse CD314 (NKG2D), anti-CD69, anti-CD25, anti-CD80, anti-CD62L, and anti-TNF-α, these antibodies were obtained from eBioscience (San Diego, CA, USA). FITC-B540-labeled anti-CD3, APC-cy7-labeled anti-NK1.1, PE-YG582-labeled anti-CTLA4, PE-cy7-YG780-labeled anti-TIGIT, APC-R660-labeled anti-PD-1, V450-labeled anti-LAG-3, Percpcy5.5-B695-labeled anti-Tim-3, YG780-labeled anti-Granzyme B, and B540-labeled anti-perforin were obtained from Biolegend (California,USA) and BD (New York, USA). For the analysis of intracellular molecules, cells stained with anti-CD4 and anti-CD8 antibodies were fixed using Fix/Perm Buffer (eBioscience, San Diego, CA, USA) for 30 min, incubated with anti-mouse-IFN-γ and -TNF-α for 30 min at 4 °C, and then analyzed using flow cytometry.

### Apoptosis assay

After being treated with STAT3 decoy ODN or scrambled ODN for 12 h, cells were harvested, washed with PBS at 4 °C, and resuspended in 100 μL binding buffer (1 × 10^6^ cells/mL) containing 5 μL of Annexin V-FITC and 10 μL of PI (propidium iodide) using an Annexin V–FITC kit (BestBio, Shanghai, China). After incubation for 10–15 min in the dark at room temperature, these cells were analyzed by flow cytometry.

### Splenocyte proliferation assay

One week after the last immunization, splenocytes were isolated from BALB/c mice and plated into 96-well plates. The splenocytes were then co-cultured with inactivated H22 cells at a ratio of 50:1. After 5 days, a 3-(4,5-dimethylthiazol-2-yl)-2,5-diphenyltetrazolium bromide (MTT) assay was performed to test the proliferation of the splenocytes.

### Tumor-reactive IgG assay

To detect tumor-reactive serum IgG, H22 cells were cultured with serum harvested from BALB/c mice immunized with HCC-vaccines for 4 h, washed thoroughly, and stained with an FITC-labeled anti-mouse IgG antibody, and analyzed by flow cytometry using a FACSCalibur flow cytometer.

### Cytotoxicity assay

H22 cells were cultured with serum harvested from HCC-vaccine immunized mice for 4 h, stained with carboxyfluorescein succinimidyl ester, washed thoroughly, and plated into 12-well plates. The splenocytes harvested from untreated mice were added at effector/target cell (E:T) ratio of 50:1. Twenty-four hours later, these cells were harvested and stained with 7-Aminoactinomycin D (7-AAD) for 15 min, and then analyzed by flow cytometry using a FACSCalibur flow cytometer.

### The transfer experiment

For the transfer experiment, 100 μL of serum harvested from HCC-vaccine immunized mice was injected (i.v.) into the healthy mice. The recipient mice were then challenged with 2 × 10^6^ H22 cells (s.c.) in their right flank at day 2. The tumor volume was measured at day 20.

### Elisa

The levels of IL-10 and TGF-β in the serum of mice were assayed by enzyme-linked immunosorbent assay (ELISA) (ExCell Bio, Shanghai, China).

### Statistical analysis

Statistical analysis was performed using a paired Student’s t test. Statistical significance was determined as ***p < 0.001, **p < 0.01 and *p < 0.05 compared with the control.

## Results

### Blockage of STAT3 signaling enhanced the immunogenicity of HCC cells and primed anti-HCC immune responses

To determine whether blocking STAT3 could increase the immunogenicity of HCC and apply a tumor vaccine against HCC, we prepared the tumor vaccine using two murine hepatoma cell lines H22 and Hepa1–6. First, HCC cells were efficiently transfected with the STAT3 decoy-ODN (Additional file 1: Fig. S1A**)**, which competitively blocked STAT3 with high specificity and promoted HCC apoptosis (Additional file 1: Fig. S1B). HCC cells treated with the scrambled-ODN or Lipofectamine were used as controls. These hepatoma cells were inactivated by repeated liquid nitrogen freeze-thawing, and the cell lysates were acquired and used as HCC vaccines; PBS was used as a blank control.

**Fig. 1:**
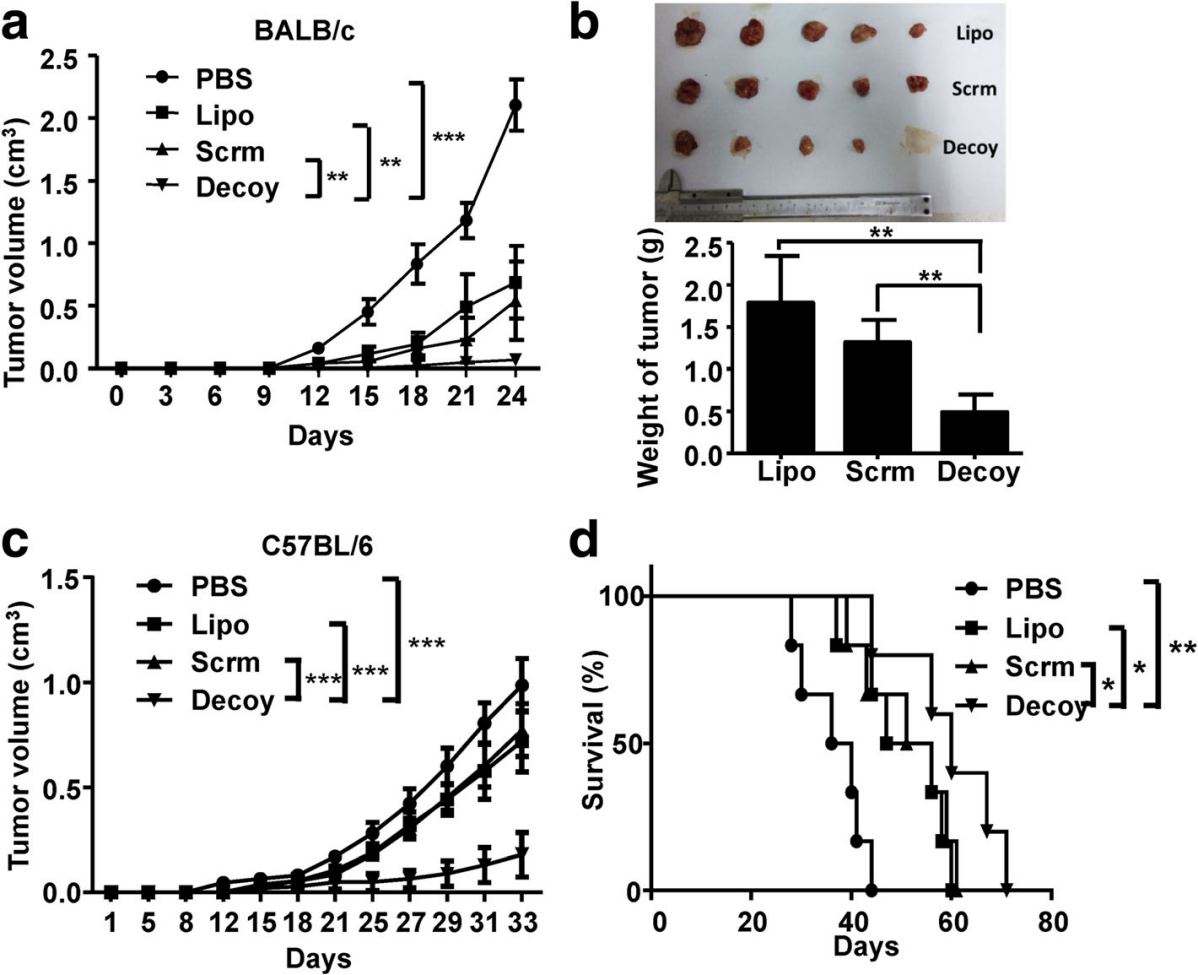
The HCC-vaccine could inhibit tumorigenesis and progression. BALB/c and C57BL/6 mice were immunized with H22- or Hepa1–6- cell derived tumor vaccine once a week for three weeks. At the first week after the last immunization, BALB/c and C57BL/6 mice were inoculated subcutaneously with 2 × 10^6^ H22 or Hepa1–6 cells, respectively. The tumor volume growth curve of tumor-bearing BALB/c (**a**) and C57BL/6 (**c**) mice were monitored. **b** Tumor size and tumor weight of BALB/c were detected at week 4, mice in the PBS group had all died within 4 weeks. **d** The survival of tumor-bearing C57BL/6 mice immunized with HCC vaccine was monitored until all the mice died. PBS, mice immunized with PBS. Lipo, Scrm, and Decoy, mice immunized with Lipo, scrambled, or Decoy-ODN-treated HCC vaccine. Data are expressed as the mean ± SD, statistical significance was determined as *p < 0.05, **p < 0.01 and ***p < 0.001 (*n* = 5)

To investigate the protective effect of the vaccines against HCC, BALB/c mice and C57BL/6 mice were immunized with the H22- or Hepa1–6-based whole cell lysate vaccine once a week for 3 weeks. These mice were then challenged with untreated-HCC cells 1 week after the last immunization, and the tumor volumes of mice were monitored ever 2–3 days. The results showed the rate of formation rate of H22 tumors in BALB/c mice immunized with STAT3-blocked HCC vaccine was 50%, which was significantly lower than that of the other groups (*p* < 0.001), while in C57BL/6 mice the tumor formation rate was not affected by immunization with Hepa1–6-based tumor vaccines (Additional file 1: Table S1). Furthermore, as shown in Fig. 1a, immunization with STAT3-blocked whole-cell hepatoma vaccine resulted in significant antitumor activity in BALB/c mice challenged with H22 tumor cells, showing significantly lower tumor growth rates compared with the other groups (Fig. 1b **)**. Meanwhile, mice in PBS group all died within 4 weeks. Similar results were observed in C57BL/6 mice challenged with Hepa1–6 cells (Fig. 1c), and the survival of mice immunized with STAT3-blocked HCC vaccine was prolonged markedly (Fig. 1d). These findings indicated that blocking STAT3 in HCC cells increased the immunogenicity of HCC.

### Immunization with STAT3-blocked whole-cell hepatoma vaccine induced the activation of T cells and NK cells

**Fig. 2:**
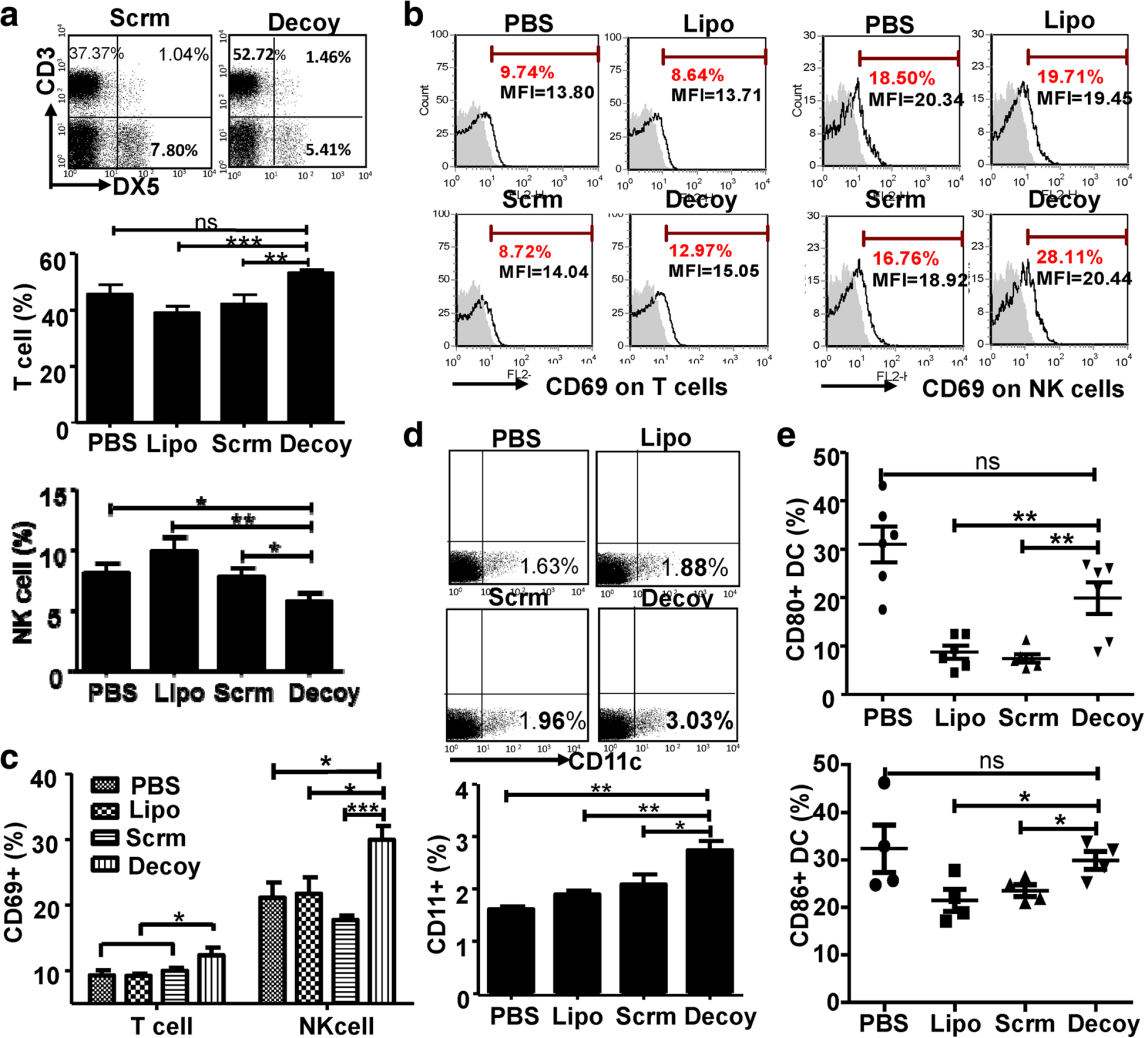
The HCC-vaccine activated the immune system in vivo. BALB/c mice were immunized with the H22 tumor vaccine once a week for three weeks. At the first week after the last immunization, PBMCs were isolated and analyzed by flow cytometry. **a** The proportion of CD3^+^DX5^−^ or CD3^−^DX5^+^ cells in PBMC of BALB/c were assayed. **b** & **c** The expression of CD69 on T cells and NK cells of BALB/c mice are shown (shaded histograms are isotype controls). **d** & **e** The proportion of CD11c^+^ DC cells and the expression of costimulatory molecules CD80 and CD86 on DCs were detected by FACS. PBS, mice immunized with PBS; Lipo, Scrm, and Decoy, mice immunized with Lipo, scrambled, or Decoy-ODN-treated HCC vaccine. Data are expressed as the mean ± SD, statistical significance was determined as *p < 0.05; **p < 0.01 and ***p < 0.001 (n ≥ 4)

To explore the potential mechanisms by which STAT3-blocked HCC vaccine displayed its anti-tumor activity, BALB/c mice were immunized with the HCC vaccine three times, and then the major anti-tumor effector cells of these mice were analyzed 1 week after the last immunization. First, we observed that the CD3^+^DX5^−^T cell population increased while the CD3^−^DX5^+^NK cell population decreased significantly in peripheral blood mononuclear cells (PBMCs) from mice immunized with STAT3-blocked HCC vaccine compared with the other groups (Fig. 2a). In contrast, in the C57BL/6 mice model, immunization with STAT3-blocked HCC vaccine did not affect the CD3^+^DX5^−^T cell population, while the CD3^−^DX5^+^NK cell population increased significantly compared with the control mice (Additional file 1: Fig. S2A). CD69 molecule, a sensitive indication of lymphocyte activation, was elevated in T cells and NK cells of PBMCs from both mouse models immunized with STAT3-blocked HCC vaccine compared with the control mice (Fig. 2c, Additional file 1: Fig. S2B). In addition, as shown in Fig. 2d **,** the proportion of CD11c^+^ Dendritic Cells (DCs) increased in mice immunized with STAT3-blocked HCC vaccine, accompanied by the upregulation of costimulatory molecules CD80 and CD86 compared with mice immunized with scrambled-ODN-treated HCC vaccine or mice injected with PBS only (Fig. 2e). These results indicated that immunization with STAT3-blocked HCC vaccine could activate the immune system.

### STAT3-blocked whole-cell hepatoma vaccine induced the generation of immune memory

**Fig. 3:**
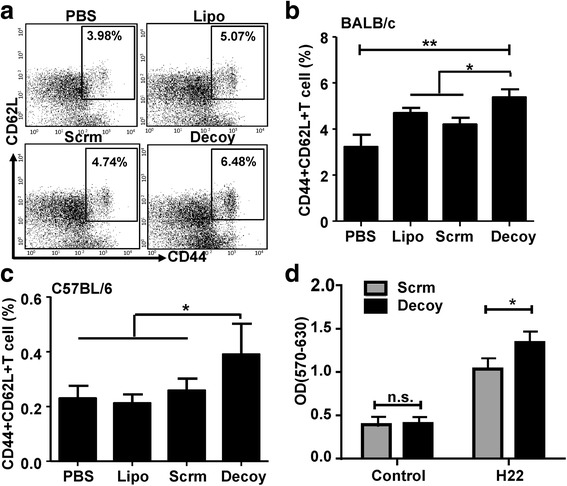
The HCC vaccine promoted the generation of immune memory. PBMCs of BALB/c mice and C57BL/6 mice were isolated at the first week after the last immunization. The proportion of CD44^+^ CD62L^+^ cells in PBMCs from BALB/c mice (**a** & **b**) and C57BL/6 mice (**c**) was detected by FACS. **d**. The splenocytes of BALB/c mice were isolated at the first week after the last immunization, and an MTT assay was used to test the proliferation of the splenocytes treated or untreated (Control) with mitomycin-inactivated H22 cells for 5 days. PBS, mice immunized with PBS; Lipo, Scrm, and Decoy, mice immunized with Lipo, scrambled, or Decoy-ODN-treated HCC vaccine. Data are expressed as the mean ± SD, statistical significance was determined as *p < 0.05 and **p < 0.01 (*n* = 6)

Immune memory plays a critical role in the anti-tumor effect of a vaccine. Memory lymphocytes, including memory T cells and memory B cells, are the executors of immune memory [17, 18]. In the present study, we observed that significantly more CD3^+^CD44^high^CD62L^+^ memory T cells were induced in PBMCs from mice immunized with STAT3-blocked HCC vaccine compared with those from the other groups (Fig. 3a & b **)**. Similar results were observed in C57BL/6 mice immunized with HCC vaccines (Fig. 3c). Furthermore, to characterize the properties of these cells, the proliferative capacity of splenocytes from immunized mice in response to H22 cells was assessed. The splenocytes from BALB/c mice immunized with STAT3-blocked (Decoy) or Scrambled ODN-treated HCC vaccine were cultured in the presence or absence of mitomycin C-treated H22 cells, respectively, for 5d in vitro, after which the proliferation of the splenocytes was detected. The results showed that the proliferative ability of splenocytes from mice immunized with STAT3-blocked HCC vaccine was significantly higher than that of the control mice (Fig. 3d). These results suggested that immunization with STAT3-blocked HCC vaccine could induce the generation of immune memory against HCC.

### STAT3-blocked whole-cell hepatoma vaccine induces a secondary immune response to HCC

**Fig. 4:**
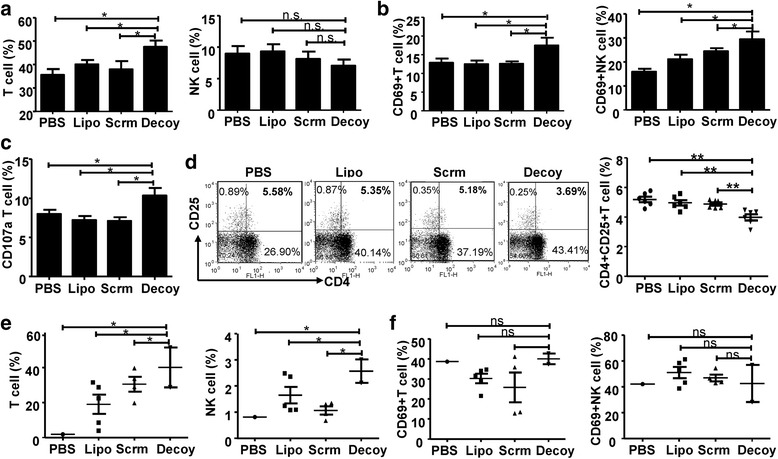
The HCC vaccine induced a secondary immune response to HCC. BALB/c mice were inoculated subcutaneously with 2 × 10^6^ tumor cells after three rounds of immunization with the HCC-vaccine, and then the PBMCs or tumor infiltrating lymphocytes of BALB/c mice were isolated when the tumor was visible. **a** The proportion of CD3^+^DX5^−^ or CD3^−^DX5^+^ cells in the PBMCs of BALB/c. **b** & **c** The expression of CD69 and CD107a on T cells or NK cells in the PBMCs of BALB/c were analyzed by flow cytometry. **d** The proportion of Tregs (CD3^+^CD4^+^CD25^+^) in the PBMCs was detected by flow cytometry. **e** & **f** The proportion and the expression of CD69 on T cells and NK cells in tumor tissues from BALB/c mice were analyzed by flow cytometry. Data are expressed as the mean ± SD, statistical significance was determined as *p < 0.05 and **p < 0.01 (*n* = 6)

BALB/c mice immunized three times with HCC vaccines as above were inoculated subcutaneously with 2 × 10^6^ H22 cells. One week later, as shown in Fig. 4a, the population of CD3^+^DX5^−^T cells from BALB/c mice immunized with STAT3-blocked HCC vaccine had increased, while there was no difference in the population of CD3^−^DX5^+^ NK cells among the different groups. Meanwhile, the expression of CD69 on both T cells and NK cells in PBMCs from mice immunized with STAT3-blocked HCC vaccine were significantly higher than those from the control mice (Fig. 4b). In addition, production of degranulation molecule CD107a was induced in T cells by the STAT3-blocked HCC vaccine (Fig. 4c). In addition, the number of CD3^+^CD4^+^CD25^+^Tregs in PBMCs from the tumor-bearing BALB/c mice immunized with STAT3-blocked HCC vaccine decreased (Fig. 4d). Similar results were shown in the C57BL/6 mice (Additional file 1: Fig. S3). One month after tumor challenge, only one mouse in the PBS group survived, whereas only two mice immunized with STAT3-blocked HCC vaccine established HCC tissue (Fig. 4e, f). Moreover, the proportion of tumor-infiltrated T cells and NK cells in mice immunized with the STAT3-blocked HCC vaccine was significantly increased compared with those in the other groups (Fig. 4e), while CD69 expression on tumor-infiltrated T cells and NK cells was not affected by immunization with the STAT3-blocked HCC vaccine (Fig. 4f). These results proved that the secondary immune response was induced in the immunized mice inoculated with the homologous tumor cells.

### The STAT3-blocked whole-cell hepatoma vaccine-induced anti-tumor effect is dependent on both cellular and humoral immunity

**Fig. 5:**
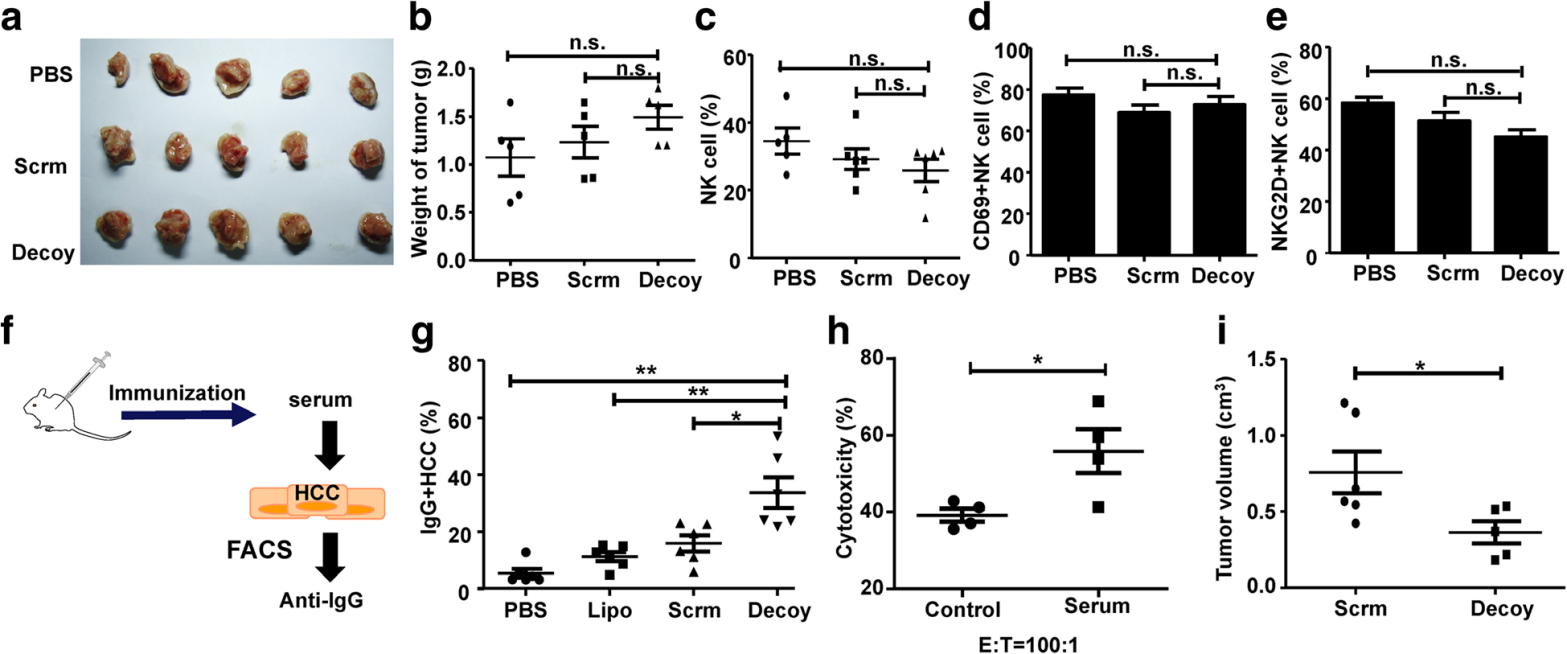
The HCC vaccine-induced anti-tumor effect was dependent on cellular and humoral immunity. Nude mice were inoculated subcutaneously with 2 × 10^6^ H22 cells after three rounds of immunization with the HCC vaccine. The tumor volume (**a**) and tumor weight (**b**) were detected at week 4. **c-e** PBMCs were isolated when the tumor was visible, and the proportion of NK cells (**c**), and the expression of CD69 (**d**) and NKG2D (**e**) on NK cells were detected by FACS. **f-h** Hepa1–6 cells were cultured with the serum from C57BL/6 mice immunized with the HCC vaccine for 4 h at 1% concentration. IgG^+^ cells were labeled by anti-mouse IgG antibody and analyzed by FACS (**g**); and, the cytotoxicity of spleen lymphocytes obtained from mice untreated with these Hepa1–6 cells was analyzed by 7-Aminoactinomycin D (7-AAD) staining methods (**h**). **i** BALB/c mice were immunized with H22 tumor vaccine once a week for three weeks. The PBMCs were isolated one week after the last immunization and transferred into healthy pre-irradiated BALB/c mice. After one week, these receptive mice were inoculated subcutaneously with 2 × 10^6^ H22 and the tumor volumes were measured after four weeks. Data are expressed as the mean ± SD, statistical significance was determined as *p < 0.05 and ** p < 0.01 (*n* = 4–6)

Previously, we confirmed that STAT3-blocked HCC could augment NK cell cytotoxicity against HCC and increase the expression of molecules associated with NK cell activation and cytotoxicity [16]. Therefore, to determine whether the STAT3-blocked HCC vaccine-induced anti-tumor immune response was mediated by NK cells, we performed an immunization strategy in nude mice that lack mature T cells. As shown in Fig. 5a-b **,** the STAT3-blocked HCC vaccine failed to delay tumor progression in the nude mice, and there was no significant difference in the tumor size and weight among the different groups. The population of NK cells, and the levels of CD69 and NKG2D on the NK cells were not changed significantly by immunization with the STAT3-blocked HCC vaccine (Fig. 5c-e). These results indicated that the STAT3-blocked HCC vaccine could not activate NK cells under deficiency of T cells. Subsequently, we explored whether humoral immunity was involved in the anti-HCC effect elicited by the STAT3-blocked HCC vaccine. We found that the levels of tumor-specific IgG antibodies from mice immunized with the STAT3-blocked HCC vaccine were significantly increased (Fig. 5f-g). Furthermore, the cytotoxicity of spleen lymphocytes against H22 cells was stronger in the presence of serum from mice immunized with the STAT3-blocked HCC vaccine, suggesting that tumor-specific IgG was essential for inhibiting tumor growth (Fig. 5h). Finally, the serum from mice immunized with the HCC vaccine was transferred to naive BALB/c mice, and these recipient mice were inoculated subcutaneously with H22 cells. The results showed that serum from mice immunized with the STAT3-blocked HCC vaccine significantly inhibited tumor growth compared with serum from the controls (Fig. 5i). These results indicated that the anti-tumor effect induced by the STAT3-blocked HCC vaccine was dependent on both cellular and humoral immunity.

### STAT3-blocked whole-cell hepatoma vaccine prevented tumor-induced exhaustion of CD8+ T and NK cells

**Fig. 6:**
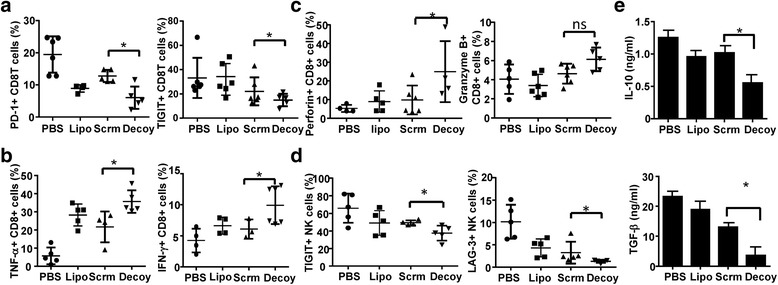
The STAT3-blocked HCC-vaccine prevented tumor-induced CD8+ T and NK cell exhaustion. BALB/c mice were inoculated subcutaneously with 2 × 10^6^ H22 cells after three rounds immunization. Four weeks later, the mice were killed when the control mice became moribund, and mononuclear cells were isolated from their spleens. **a** The levels of PD-1, CTLA-4, TIM-3, TIGIT, and LAG-3 were detected by FACS on T cells from the different groups. **b** Intracellular TNF-α and IFN-γ of CD4^+^ T cells were detected by FACS. c The percentage of Granzym B and perforin positive CD8^+^ T cells were assayed by FACS. **d** The levels of PD-1, CTLA-4, TIM-3, TIGIT and LAG-3 on NK cells was analyzed by FACS. **e** The levels of IL-10 and TGF-β in the serum of mice were assayed by enzyme-linked immunosorbent assay (ELISA). Data are expressed as the mean ± SD, statistical significance was determined as *p < 0.05 and **p < 0.01 (n = 6)

Previous studies demonstrated that T cell dysfunction or exhaustion in tumor-bearing mice would hinder the production of anti-tumor immunity. To date, (Programmed Death 1) PD-1, cytotoxic T-lymphocyte antigen 4 (CTLA-4), T-cell immunoglobulin mucin-3 (TIM-3), T-cell immunoreceptor with Ig and ITIM domains (TIGIT), and Lymphocyte-activation gene 3 (LAG-3) have been identified as markers of exhausted T cells or NK cells [19–21]. To examine the potential role for preventing CD8^+^ T and NK cell exhaustion by the STAT3-blocked HCC vaccine, we first examined the expression of these exhaustion markers on T cells and NK cells from mice bearing H22 cells. We observed that the number of PD-1^+^CD8^+^ T cells and TIGIT^+^CD8^+^ T cells was lower in H22-bearing mice immunized with the STAT3-blocked HCC vaccine than in the other groups, while LAG-3 and TIM-3 expression did not show significant differences (Fig. 6a). We then examined the production of Tumor Necrosis Factor α (TNF-α) and Interferon γ (IFN-γ) to determine whether CD8^+^ T cells exhibited an exhausted phenotype. We found that the CD8^+^ T cells displayed the most profound impairment in production of IL-2 (data not shown); however, the STAT3-blocked HCC vaccine improved the production of TNF-α and IFN-γ by CD8^+^T cells (Fig. 6b), as well as the percentage of perforin-expressing CD8^+^T cells. A similar trend was observed with Granzyme B, although this did not reach statistical significance (Fig. 6c). Meanwhile, as shown in Fig. 6d **,** LAG-3 and TIGIT, but not PD-1, LAG-3, or CTLA-4, were lower on NK cells from mice immunized with the STAT3-blocked HCC vaccine. Finally, the levels of IL-10 and TGF-β in mice immunized with the STAT3-blocked HCC vaccine were lower than those in the other groups (Fig. 6e). These results indicated that the STAT3-blocked HCC vaccine could prevent CD8^+^ T cells and NK cells from HCC-induced exhaustion.

## Discussion

Previous studies have suggested that whole-cell tumor vaccines are more effective clinically when combined with modalities, such as treatment with soluble cytokines, immunomodulatory drugs, or anti-angiogenics, chemotherapy, and radiotherapy [22]. In the current study, we expanded the scope of whole-cell tumor vaccines. We prepared whole HCC cell lysates from HCC cells in which the STAT3 signaling pathway was blocked. The lysates acted as whole-cell HCC vaccines and resulted in inhibition of tumor growth and increased survival of mice challenged with HCC (Fig. 1). We observed that the tumor formation rate and immune cell activation were different between C57BL/6 mice and BALB/c mice (Fig. 1 & Additional file 1: Table S1), which might be associated with the differences in the immune background, immunogenicity, and immune status of these two different murine strains [23, 24].

STAT3 is overexpressed in more than 90% of human carcinomas, including the breast, lung, colorectal, and hepatoma [25]. High levels of STAT3 are associated with aggressive progression, metastasis, and recurrence of liver cancer [26]. Previously, we proved that abrogation or inhibition of STAT3 attenuated tumor growth in vitro and in vivo [15, 16]. Interestingly, NK cell-mediated anti-tumor effects were induced by inoculation with STAT3-blocked HCC cells in mice [16], indicating that blocking STAT3 would augment the immunogenicity of HCC cells, in addition to reversing HCC-mediated immune suppression. Indeed, the prepared HCC vaccine could induce the activation of T cells and NK cells in vivo, as well as the maturation of CD11c^+^ DC cells (Fig. 2). Importantly, the STAT3-blocked whole-cell HCC vaccine promoted the generation of immune memory against HCC (Fig. 3). To explain why the immunogenicity of whole HCC cell lysates was changed by STAT3-blockage, gene array analysis was performed. The data showed that the expression profiles of genes encoding chemokines, molecules associated cell proliferation, and inflammatory molecules in the HCC cell lysates were altered by STAT3-blockage (Additional file 1: Fig. S3). It is likely that the levels of the proteins encoded by these genes would be changed too; however, the exact components that contribute to the observed immunogenicity need to be further studied.

Currently, many studies on cancer immunotherapy have focused on immunosuppressive markers, targets, and combinational approaches, and have demonstrated that priming the anti-tumor immunity by dampening the tumor immunosuppressive environment might be an effective approach for cancer treatment [27, 28]. The present study showed that the secondary immune response was triggered in HCC vaccine-immunized mice challenged with the homologous tumor cells, accompanied by the activation of T cells and NK cells, the elimination of Tregs, and enhanced recruitment of CD8^+^ T cells in tumor tissues (Fig. 4). Further investigation showed that T cells were required for the anti-tumor response elicited by the HCC vaccine. In addition, the cytotoxic effects mediated by splenocytes from HCC vaccine-immunized mice were significantly enhanced against target cells. Simultaneously, the HCC vaccine could induce the production of tumor specific-IgG, which also mediated the anti-HCC immune response (Fig. 5).

Tumor cells can directly escape from T-cell recognition by downregulating MHC class I but upregulating surface ligands, such as PD-L1, CTLA-4, and certain other ligands of inhibitory T-cell receptors, which mediate T-cell exhaustion [20, 29, 30]. Chakrabarti demonstrated that an attenuated Id2-kd whole-cell neuroblastoma vaccine was safe in mice and could induce a broad tumor-specific cellular immunity, which protected against tumor formation in prophylactic tumor models and eradicated large established neuroblastoma tumors in combination with an anti-CTLA-4 antibody [31]. A recent study showed that the IL-27/STAT3 axis induced the expression of PD-L1/2, and that STAT3 blockage reversed T cell exhaustion [32]. Furthermore, in a murine breast cancer model, a cancer vaccine therapy employing the systemic delivery of a tumor-targeting Salmonella-based STAT3 shRNA increased the proliferation and granzyme B levels of intratumoral CD4^+^ and CD8^+^ T cells, which favored the destruction of malignant cells [33]. Interestingly, in our study, we also observed that the STAT3-blocked HCC vaccine could prevent tumor-induced exhaustion of CD8^+^ T and NK cells, as depicted by the downregulation of PD-1, TIGIT, and LAG-3 in HCC-vaccine-immunized mice (Fig. 6).

An effective therapy for HCC is the ultimate goal of researchers because of the poor prognosis for patients with HCC. A vaccine that increased immunogenesis and reset the immune-suppressive environment would very likely result in ideal clinical outcomes with favorable effects for patients with HCC. The STAT3-blocked whole-cell HCC vaccine disclosed in the present study displayed several attractive characteristics. It could achieve maximal anti-tumor effects by augmenting the activation of both T cells and NK cells, as well as the production of antibodies. Importantly, the STAT3-blocked whole-cell HCC vaccine protected against the exhaustion of T cells and NK cells, facilitating a secondary immune response against HCC. In addition to vaccination against HCC, this strategy might also be applicable to other types of cancer that involve STAT3 over-activation. Further studies are required to determine the molecular mechanisms by which the STAT3-blocked HCC vaccine promotes the crosstalk between STAT3 in tumor cells and immune cells, and relevant techniques before its use in clinical trials. However, our study provided support for the clinical use of genetically modified tumor cells as cancer vaccines.

## Conclusion

The STAT3-blocked whole-cell hepatoma vaccine can augment the cellular and humoral immune responses against HCC, resulting the inhibition of tumor growth and tumorigenesis. Thus,the newly generated STAT3-blocked whole-cell HCC vaccine displayed the potential for cancer cell vaccinations.

## Additional file

Additional file 1:
**Table S1.**The tumor formation rate in mice immunized with HCC-vaccine. **Figure S2.** HCC-vaccine can activate immune system in C57BL/6 mice. **Figure S1.** Decoy ODN were effectively transfected into HCC cells; **Figure S3.** HCC vaccine induced the secondary immune response to HCC in C57BL/6 mouse model. **Figure S4.** mRNA levels of chemockines, and molecules associated cell proliferation and inflammation are regulated by STAT3 blocking. (PDF 901 kb)

## Abbreviations

7-AAD: 7-Aminoactinomycin D
APC: Allophycocyanin
CTL: CD8^+^ cytotoxic lymphocytes
CTLA-4: Cytotoxic T-lymphocyte antigen 4
DC: Dendritic Cells
DMEM: Dulbecco’s modified Eagle’s medium
ELISA: Enzyme-linked immunosorbent assay
FAP: Fibroblast activation protein
FBS: Fetal bovine serum
FITC: Fluorescein isothiocyanate
GM-CSF: Granulocyte-macrophage colony-stimulating factor
HCC: Hepatocellular carcinoma
IFN-γ: Interferon γ
LAG-3: Lymphocyte-activation gene 3
MAGE-A: Melanoma-associated antigen
MTT: 3-(4,5-dimethylthiazol-2-yl)-2,5-diphenyltetrazolium bromide
NK cell: Natural killer cell
NY-ESO-1: New York esophageal squamous cell carcinoma 1
ODN: Oligodeoxynucleotide
PBMCs: Peripheral blood mononuclear cells
PBS: Phosphate-buffered saline
PD-1: Programmed death 1
PE: Phycoerythrin
PI: Propidium iodide
RPMI: Roswell Park Memorial Institute
SSX-2: Synovial sarcoma, X Breakpoint 2
STAT3: Signal transducer and activator of transcription 3
TIGIT: T cell immunoglobulin and immunoreceptor tyrosine-based inhibitory motif [ITIM] domain
Tim-3: T-cell immunoglobulin mucin-3
TNF-α: Tumor Necrosis Factor α

## References

[1] Tagliamonte M, Petrizzo A, Tornesello ML, Ciliberto G, Buonaguro FM, Buonaguro L (2016). Combinatorial immunotherapy strategies for hepatocellular carcinoma. Curr Opin Immunol.

[2] Mikhail S, Cosgrove D, Zeidan A (2014). Hepatocellular carcinoma: systemic therapies and future perspectives. Expert Rev Anticancer Ther.

[3] Buonaguro L, Petrizzo A, Tagliamonte M, Tornesello ML, Buonaguro FM (2013). Challenges in cancer vaccine development for hepatocellular carcinoma. J Hepatol.

[4] Greten TF, Forner A, Korangy F, N'Kontchou G, Barget N, Ayuso C, Ormandy LA, Manns MP, Beaugrand M, Bruix J (2010). A phase II open label trial evaluating safety and efficacy of a telomerase peptide vaccination in patients with advanced hepatocellular carcinoma. BMC Cancer.

[5] Saini N, Srinivasan R, Chawla Y, Sharma S, Chakraborti A, Rajwanshi A (2009). Telomerase activity, telomere length and human telomerase reverse transcriptase expression in hepatocellular carcinoma is independent of hepatitis virus status. Liver Int.

[6] Tada F, Abe M, Hirooka M, Ikeda Y, Hiasa Y, Lee Y, Jung NC, Lee WB, Lee HS, Bae YS (2012). Phase I/II study of immunotherapy using tumor antigen-pulsed dendritic cells in patients with hepatocellular carcinoma. Int J Oncol.

[7] Chiang CL, Coukos G, Kandalaft LE (2015). Whole tumor antigen vaccines: where are we?. Vaccines (Basel).

[8] Papaioannou NE, Beniata OV, Vitsos P, Tsitsilonis O, Samara P (2016). Harnessing the immune system to improve cancer therapy. Ann Transl Med.

[9] Kajihara M, Takakura K, Ohkusa T, Koido S (2015). The impact of dendritic cell-tumor fusion cells on cancer vaccines - past progress and future strategies. Immunotherapy.

[10] Copier J, Dalgleish A (2010). Whole-cell vaccines: a failure or a success waiting to happen?. Curr Opin Mol Ther.

[11] Zhuang XB, Xing N, Zhang Q, Yuan SJ, Chen W, Qiao TK (2015). CpG Oligodeoxynucleotide1826 combined with radioresistant cancer cell vaccine confers significant antitumor effects. Neoplasma.

[12] Chen M, Xiang R, Wen Y, Xu G, Wang C, Luo S, Yin T, Wei X, Shao B, Liu N (2015). A whole-cell tumor vaccine modified to express fibroblast activation protein induces antitumor immunity against both tumor cells and cancer-associated fibroblasts. Sci Rep.

[13] Fishman M, Hunter TB, Soliman H, Thompson P, Dunn M, Smilee R, Farmelo MJ, Noyes DR, Mahany JJ, Lee JH (2008). Phase II trial of B7-1 (CD-86) transduced, cultured autologous tumor cell vaccine plus subcutaneous interleukin-2 for treatment of stage IV renal cell carcinoma. J Immunother.

[14] Yu H, Lee H, Herrmann A, Buettner R, Jove R (2014). Revisiting STAT3 signalling in cancer: new and unexpected biological functions. Nat Rev Cancer.

[15] Sun XX, Sui QJ, Zhang C, Tian ZG, Zhang J (2013). Targeting blockage of STAT3 in Hepatocellular carcinoma cells augments NK cell functions via reverse Hepatocellular carcinoma-induced immune suppression. Mol Cancer Ther.

[16] Sui QJ, Zhang J, Sun XX, Zhang C, Han QJ, Tian ZG (2014). NK cells are the crucial antitumor mediators when STAT3-mediated Immunosuppression is blocked in Hepatocellular carcinoma. J Immunol.

[17] Lugli E, Hudspeth K, Roberto A, Mavilio D (2016). Tissue-resident and memory properties of human T-cell and NK-cell subsets. Eur J Immunol.

[18] Cerwenka A, Lanier LL (2016). Natural killer cell memory in infection, inflammation and cancer. Nat Rev Immunol.

[19] Anderson AC, Joller N, Kuchroo VK (2016). Lag-3, Tim-3, and TIGIT: co-inhibitory receptors with specialized functions in immune regulation. Immunity.

[20] Schildberg FA, Klein SR, Freeman GJ, Sharpe AH (2016). Coinhibitory pathways in the B7-CD28 Ligand-receptor family. Immunity.

[21] Dillman RO (2016). Is there a role for therapeutic cancer vaccines in the age of checkpoint inhibitors?. Hum Vaccin Immunother.

[22] Allam AB, von Chamier M, Brown MB, Reyes L: Immune profiling of BALB/C and C57BL/6 mice reveals a correlation between Ureaplasma parvum-induced fetal inflammatory response syndrome-like pathology and increased placental expression of TLR2 and CD14. Am J Reprod Immunol 2014, 71(3):241-251. 10.1111/aji.12192 PMC3927638 24372928

[23] Pascuan CG, Uran SL, Gonzalez-Murano MR, Wald MR, Guelman LR, Genaro AM (2014). Immune alterations induced by chronic noise exposure: comparison with restraint stress in BALB/c and C57Bl/6 mice. J Immunotoxicol.

[24] Seledtsov VI, Goncharov AG, Seledtsova GV (2015). Multiple-purpose immunotherapy for cancer. Biomed Pharmacother.

[25] Chai EZ, Shanmugam MK, Arfuso F, Dharmarajan A, Wang C, Kumar AP, Samy RP, Lim LH, Wang L, Goh BC (2016). Targeting transcription factor STAT3 for cancer prevention and therapy. Pharmacol Ther.

[26] He G, Karin M (2011). NF-kappaB and STAT3 - key players in liver inflammation and cancer. Cell Res.

[27] Marabelle A, Kohrt H, Sagiv-Barfi I, Ajami B, Axtell RC, Zhou G, Rajapaksa R, Green MR, Torchia J, Brody J (2013). Depleting tumor-specific Tregs at a single site eradicates disseminated tumors. J Clin Invest.

[28] Mullard A (2016). The cancer vaccine resurgence. Nat Rev Drug Discov.

[29] Liu Y, Cao X (2015). Intratumoral dendritic cells in the anti-tumor immune response. Cell Mol Immunol..

[30] Romanets-Korbut O, Kovalevska LM, Seya T, Sidorenko SP, Horvat B (2016). Measles virus hemagglutinin triggers intracellular signaling in CD150-expressing dendritic cells and inhibits immune response. Cell Mol Immunol.

[31] Chakrabarti L, Morgan C, Sandler AD (2015). Combination of Id2 knockdown whole tumor cells and checkpoint blockade: a potent vaccine strategy in a mouse Neuroblastoma model. PLoS One.

[32] Hasita H, Ma C, Yano H, Pan C, Ohnishi K, Fujiwara Y, Endo S, Kikukawa Y, Okuno Y, Matsuoka M (2016). An IL-27/Stat3 axis induces expression of programmed cell death 1 ligands (PD-L1/2) on infiltrating macrophages in lymphoma. Cancer Sci.

[33] Manuel ER, Blache CA, Paquette R, Kaltcheva TI, Ishizaki H, Ellenhorn JD, Hensel M, Metelitsa L, Diamond DJ (2011). Enhancement of cancer vaccine therapy by systemic delivery of a tumor-targeting salmonella-based STAT3 shRNA suppresses the growth of established melanoma tumors. Cancer Res.

