# Supplementary material for: STAT3-blocked whole-cell hepatoma vaccine induces cellular and humoral immune response against HCC
Source: J Exp Clin Cancer Res. 2017 Nov 7;36:156. doi: 10.1186/s13046-017-0623-0 (PMC5688805; doi:10.1186/s13046-017-0623-0)
Supplement: Additional file 1: — Table S1.The tumor formation rate in mice immunized with HCC-vaccine. Figure S2. HCC-vaccine can activate immune system in C57BL/6 mice. Figure S1. Decoy ODN were effectively transfected into HCC cells; Figure S3. HCC vaccine induced the secondary immune response to HCC in C57BL/6 mouse model. Figure S4. mRNA levels of chemockines, and molecules associated cell proliferation and inflammation are regulated by STAT3 blocking. (PDF 901 kb) [file 13046_2017_623_MOESM1_ESM.pdf]

Table S1 The tumor formation rate in mice immunized with decoy

	BALB/c		C57BL/6	
	**Number**	**Rate**	**Number**	**Rate**
**PBS**	18/18	100%	12/12	100%
**Lipo**	17/18	94%	12/12	100%
**Scrm**	15/18	83%	12/12	100%
**Decoy**	9/18	50%	12/12	100%

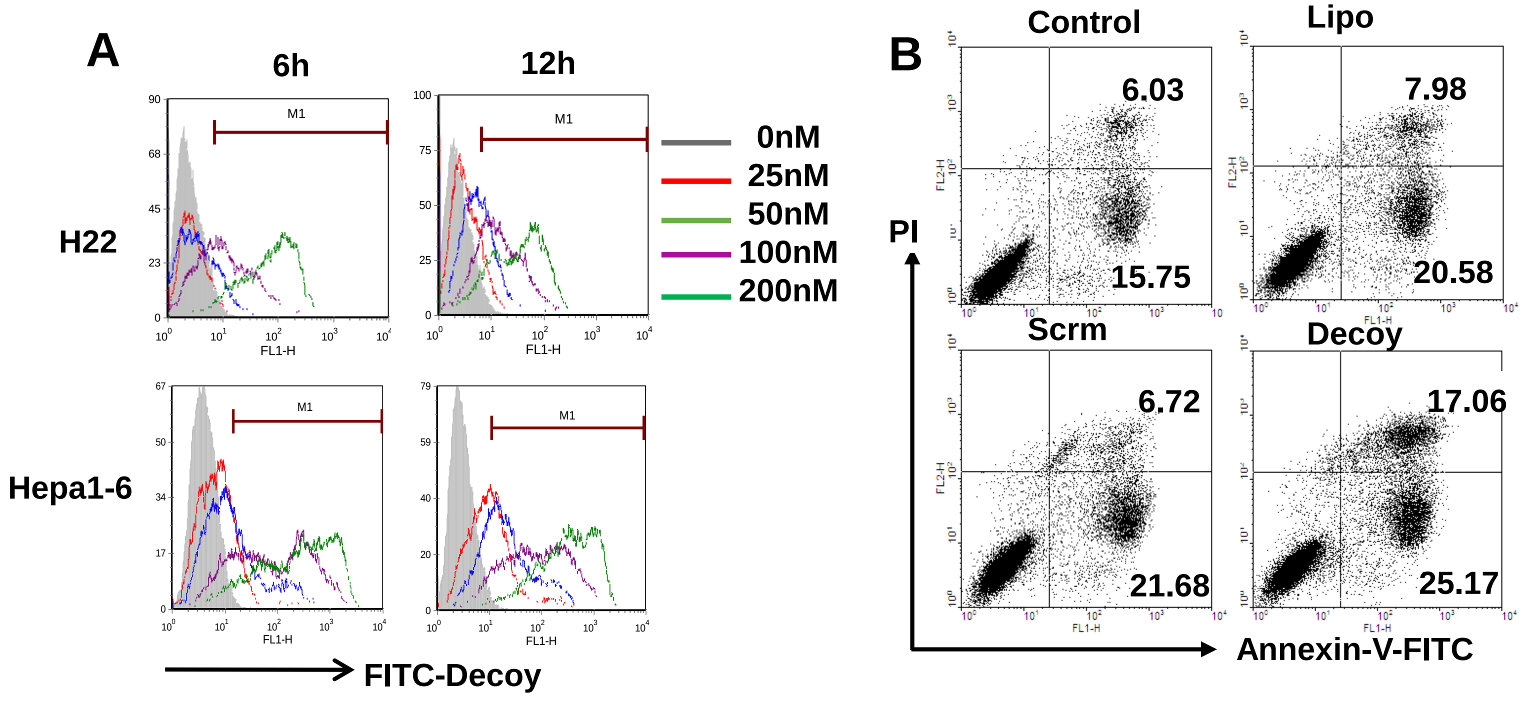

Figure S1. Decoy ODN were effectively transfected into HCC cells and functional. A. H22 cells and Hepa1-6 cells were transfected with FITC-labeled STAT3 Decoy ODN at final concentration of 25, 50, 100, and 200 nM by Lipofectamine™ 2000. After transfected for 6 h or 12 h, the transfection efficiency was examined by flow cytometry. **B.** H22 cells were transfected with 100 nM Decoy ODN for 12 h and then cultured in complete medium for 24h, the apoptosis rates were detected by Flow cytometry using Annexin V-FITC and PI. One representative of at least three independent experiments.

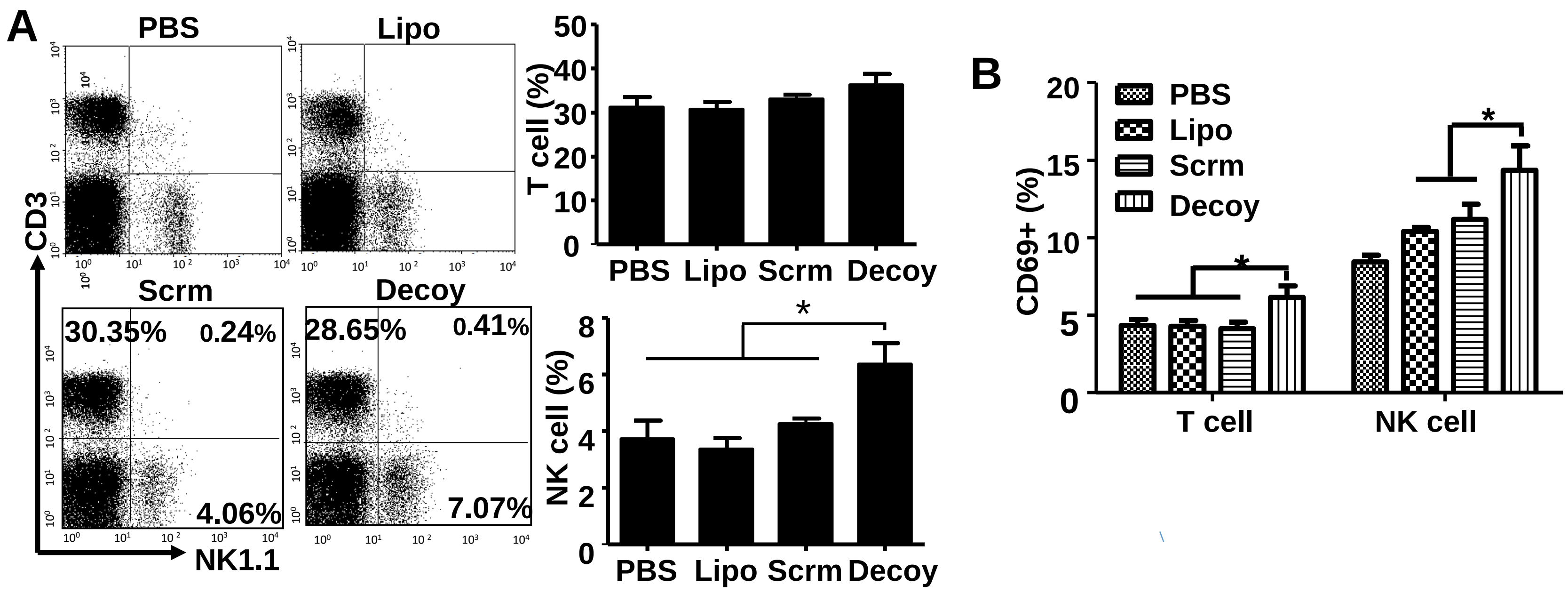

Figure S2. HCC-vaccine can activate immune system in C57BL/6 mice. C57BL/6 mice were immunized with Hepa1-6 tumor vaccine every week for three times. At the first week after the last immunization, the PBMCs were isolated and analyzed by flow cytometry. **A**. The proportion of $CD3^{+}NK1.1^{-}$ or $CD3^{-}NK1.1^{+}$ cells in PBMC of C57BL/6 mice. **B**. The expression of CD69 on T cells and NK cells of C57BL/6 mice were showed. Data were expressed as the mean ± SD, statistical significance was determined as *p<0.05; **p<0.01 and ***p<0.001 (n≥4).

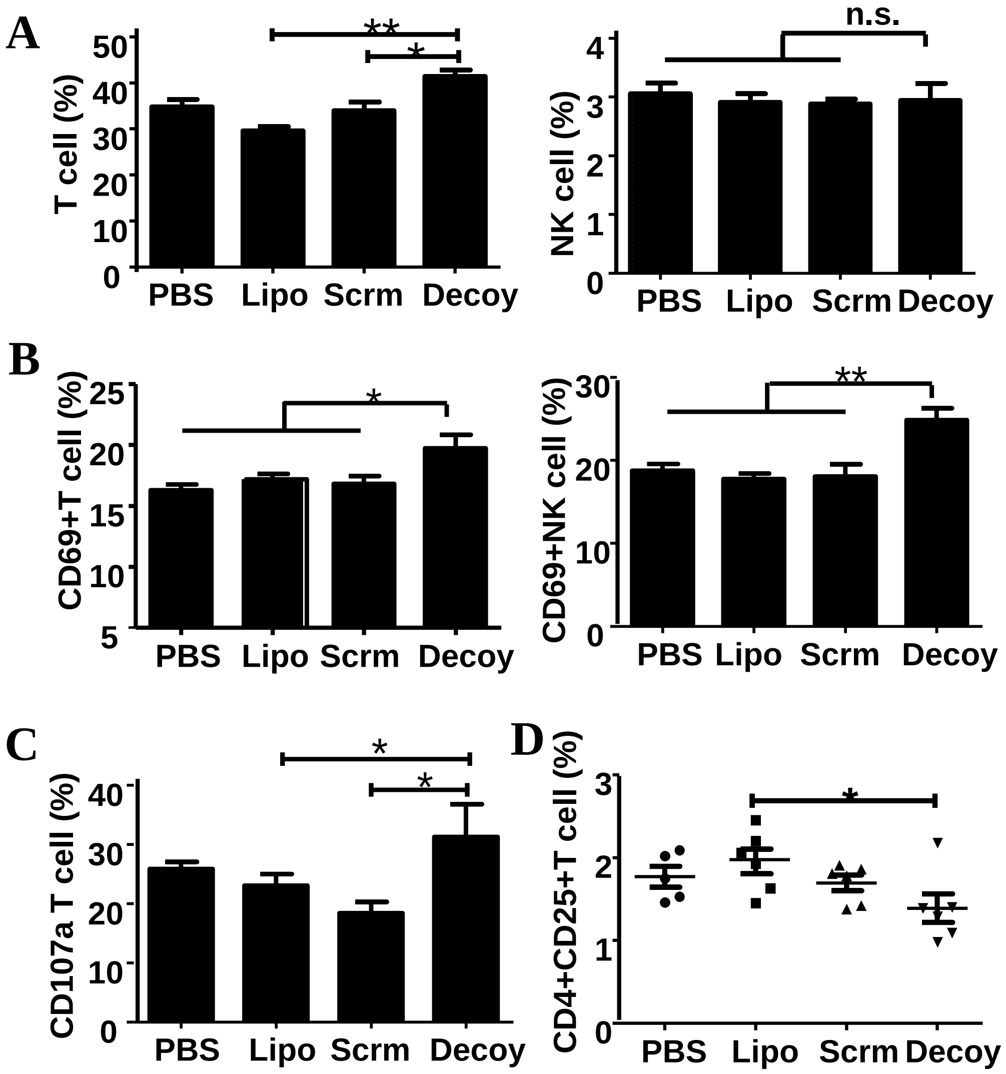

Fig. S3. HCC vaccine induced the secondary immune response to HCC in C57BL/6 mouse model. C57BL/6 mice were inoculated subcutaneously with 2 × 10^6 tumor cells after 3 times immunization with HCC-vaccine, and then the PBMC of C57BL/6 mice were isolated when the

tumor was visible. **A**.The proportion of $CD3^+DX5^-$ or $CD3^-DX5^+$ cells in PBMC of C57BL/6 mice was assayed. The expression of CD69 (**B**) and CD107a (**C**) on T cells or NK cells in PBMC of C57BL/6 mice were analyzed by Flow cytometry. **D.** The proportion of Treg ($CD3^+CD4^+CD25^+$) in PBMC was detected by Flow cytometry. Data were expressed as the mean ± SD, statistical significance was determined as *p<0.05 and **p<0.01 (n=6).

A **Hep1-6**

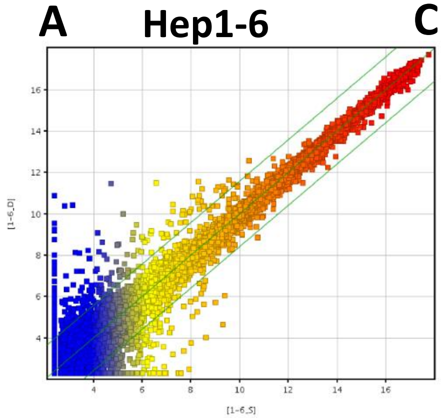

B **H22**

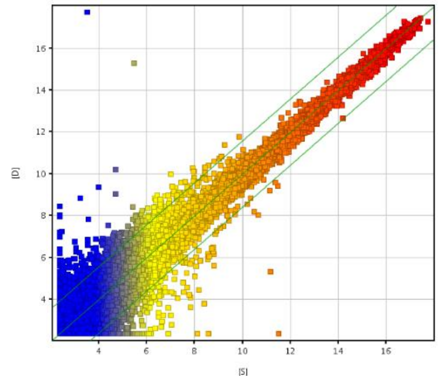

C

1-6_D vs 1-6_S 3.0 fold up regulated gene

Probe Name	...	Absolute Fold change([1-6_D] vs [1-6_S])	Regulation([1-6_D] vs [1-6_S])	...	GeneSymbol	...	[1-6_D](normalized)	[1-6_S](normalized)
A_51_P217463	...	5.479761	up	...	Cxcl2	...	8.273031	5.818918
A_51_P226711	...	3.4720674	up	...	Ptf1a	...	6.1224546	4.3266597
A_55_P2318090	...	4.237447	up	...	Amy2a4	...	6.4090605	4.3258653
A_66_P128537	...	11.624373	up	...	Isg15	...	8.541105	5.002024
A_52_P554267	...	4.6583853	up	...	Ilf3	...	4.5866094	2.3667793
A_55_P2165199	...	5.675278	up	...	Cxcr6	...	6.5868196	4.0821285
A_55_P2158741	...	3.1114335	up	...	Nos2	...	6.603411	4.9658318
...	...	...	...	...	...	...	...	...

1-6_D vs 1-6_S 3.0 fold down regulated gene

ProbeName	...	Absolute Fold change([1-6_D] vs [1-6_S])	Regulation([1-6_D] vs [1-6_S])	...	GeneSymbol	...	[1-6_D](normalized)	[1-6_S](normalized)
A_51_P253897	...	3.183136	down	...	psca	...	4.8587265	6.5291753
A_55_P2126363	...	4.24935	down	...	Wnt10b	...	6.3960934	8.4833355
A_55_P2185484	...	4.4730525	down	...	Zfp865	...	4.5642867	6.7255464
A_55_P2079079	...	3.1190076	down	...	Cd19	...	4.7175875	6.3586745
A_52_P436628	...	3.1515846	down	...	Vegfb	...	6.6709394	8.327017
A_55_P1991555	...	8.973513	down	...	Mrgprx1	...	3.025877	6.19155
A_55_P2129955	...	6.320447	down	...	Olfr1313	...	3.2448087	5.904835
...	...	...	...	...	...	...	...	...

Fig. S4. Genes encoding chemokines, molecules associated cell proliferation, and inflammatory molecules are regulated by STAT-3 blocking. H22 cells and Hepa1-6 cells were transfected with STAT3 Decoy ODN at final concentration of 100nM by Lipofectamine™ 2000. After transfected for 24h, total RNA from each sample was amplified and transcribed into fluorescent cRNA with using the manufacturer's Agilent's Quick Amp Labeling protocol (version 5.7, Agilent Technologies). The labeled cRNAs were hybridized onto the Whole Mouse Genome Oligo Microarray (4x44K, Agilent Technologies). After having washed the slides, the arrays were scanned by the Agilent Scanner G2505C. Representatives of 3.0 fold up or down regulated gene were shown when STAT3 blocking or not.1-6_D represents Hepa1-6 cells transfected with STAT3 decoy, while 1-6_S represents Hepa1-6 cells transfected with Scramble ODN.
